# Examining Self-Disclosure on Social Networking Sites: A Flow Theory and Privacy Perspective

**DOI:** 10.3390/bs8060058

**Published:** 2018-06-06

**Authors:** George Oppong Appiagyei Ampong, Aseda Mensah, Adolph Sedem Yaw Adu, John Agyekum Addae, Osaretin Kayode Omoregie, Kwame Simpe Ofori

**Affiliations:** 1 Department of Management, Ghana Technology University College, Accra PMB 100, Ghana; gampong@gtuc.edu.gh; 2 Department of Marketing and Entrepreneurship, University of Ghana Business School, Accra LG78, Ghana; 3 Department of Computer Science, Ho Technical University, Ho HP217, Ghana; aadu@htu.edu.gh; 4 Department of Finance and Accounting, Ghana Technology University College, Accra PMB 100, Ghana; jagyekum@gtuc.edu.gh; 5 Lagos Business School, Pan-Atlantic University, Lagos, Nigeria; komoregie@lbs.edu.ng

**Keywords:** self-disclosure, social networking sites, flow, privacy concerns, structural equation modeling, Ghana

## Abstract

Social media and other web 2.0 tools have provided users with the platform to interact with and also disclose personal information to not only their friends and acquaintances but also relative strangers with unprecedented ease. This has enhanced the ability of people to share more about themselves, their families, and their friends through a variety of media including text, photo, and video, thus developing and sustaining social and business relationships. The purpose of the paper is to identify the factors that predict self-disclosure on social networking sites from the perspective of privacy and flow. Data was collected from 452 students in three leading universities in Ghana and analyzed with Partial Least Square-Structural Equation Modeling. Results from the study revealed that privacy risk was the most significant predictor. We also found privacy awareness, privacy concerns, and privacy invasion experience to be significant predictors of self-disclosure. Interaction and perceived control were found to have significant effect on self-disclosure. In all, the model accounted for 54.6 percent of the variance in self-disclosure. The implications and limitations of the current study are discussed, and directions for future research proposed.

## 1. Introduction

An increasingly connected world has fast-forwarded the rate of information transfer across the globe. Social media and other web 2.0 tools have provided the platform for individuals to communicate simultaneously not only with their friends and acquaintances but also with relative strangers [1] with unprecedented ease. This has facilitated the ability of people to share more about themselves, their families, and their friends through a variety of media including text, photo, and video [2], thus developing and sustaining social and business relationships [3,4]. Ellison and Boyd [5] defined a social networking site as “a networked communication platform in which participants (1) have uniquely identifiable profiles that consist of user-supplied content, content provided by other users and/or system-level data, (2) can publicly articulate connections that can be viewed and traversed by others, and (3) can consume, produce, and/or interact with streams of user generated content provided by their connections on the site.” In the literature, this process of “making the self known to others,” described as self-disclosure [6] has been in existence at least since the late 1950s for example Jourard & Lasakow [7], and is a well-established phenomenon in the field of psychology [8]. With the introduction of social media, in particular, social networking sites, people have been found to reveal more and more of themselves online than they ordinarily would in traditional/off-line settings [6,9]. This has been attributed to the lack of some social cues that are easily evident in face-to-face communications [6], which frees individuals up to express themselves without fear or favor, as well as the absence of self-consciousness that would be existent in personal communications but is not present in online environments [10].

While current research has weighed the costs and benefits of such self-disclosure on social networking sites, citing issues of privacy [4,11], and how much it should be controlled [12], as well as considered the qualities of various social networking sites which encourage self-disclosure [3,13], there is still a limited work on the antecedents and motivators of self-disclosure from the perspective of privacy and flow. Extant studies on the topic include research by Cheung et al. [9], Krasnova et al. [11], and Walrave et al. [14]; however, these have each studied only a few variables limited to a particular social networking site like Facebook. The current study therefore aims to examine the antecedents of self-disclosure from the perspective of privacy related constructs and flow in order to answer the research question: “What factors motivate users to disclose information about themselves on social networking sites?”

The findings from such a study would fill the gaps in literature by providing empirical evidence for constructs that affect consumers’ self-disclosure on social media. It would also be of benefit to marketing practitioners, who can thus discover which factors encourage their consumers to share more about themselves and about brands on their social networking site accounts. Succeeding sections in the current paper will present a brief literature review on self-disclosure within the social networking site sphere, followed by the development of the research model and the hypotheses to be tested. The methodology, results, and discussions of the study will next be shared. The paper will conclude with recommendations to practitioners and for future research based on the findings of the study.

## 2. Literature Review

The importance of computers and technology as a mediator between two communicating parties is no longer as much of a novelty as it was just two decades ago. The unique features of web 2.0, and its role in making the internet a more accessible and interactive environment [9] even for non-technical users, have facilitated the growth and spread of social media [15]. This online space has provided rich opportunities for brands seeking to connect with their consumers, as the number of useful platforms for such interactions have ballooned over the years since the technology was introduced [16]. One of the most ubiquitous and successful applications for this purpose is social media, which has been defined as “a variety of new sources of online information that are created, initiated, circulated and used by consumers intent on educating each other about products, brands, services, personalities, and issues” [17]. Although this may take a number of forms, the most common among users by far are social networking sites, which have boasted an excess of billions of users across the globe [18]. Most popular among these are sites like Facebook, Instagram, Twitter, and MySpace, whose popularity has been attributed to the fact that not only do they allow users to connect with offline friends, but they also facilitate the creation of new friends and connections [19]. These sites also provide an opportunity for users to share their lives with those in their networks [14], using photos, text, or video [20]. This has given rise to the discussion of self-disclosure on social networking sites.

### Self-Disclosure on SNS

As social networking sites (SNS) have become more and more integrated into the lives of users, individuals share an incredible amount of information about themselves on the platforms. For example, the public presentation of personal profiles, photos, and videos [20], as well as regular status updates on daily events, preferences, experiences, and places in the lives of users [2] has made it relatively easy to discover previously private information about people with a basic Google search. It is little wonder then that some of the major discussions regarding the issue have centered on privacy concerns [4,21,22], often linked to the carelessness and/or avarice of corporations (Chen and Sharma [23]) who gain and share the private information of users once they sign up on the platform. Moreover, phenomena like cyber-bullying (sometimes leading to psychological trauma) [24], stolen data [21], and the endangerment of minors [25] have all been reported on social platforms, such that several bodies have called for tighter monitoring of SNSs.

However, consumers themselves seem undeterred by the dark side of social media. Researchers have observed that people are still more open on such platforms where there is a sense of anonymity [26] and freedom of self-expression [6], which may not be the case in face-to-face conversations. According to Meeker (2014), approximately 1.8 billion personal photographs are shared daily across the five leading SNS. The reasons for such overwhelming self-disclosure through both verbal and non-verbal channels [20] has been queried by scholars, who find that users seek to maintain their social bonds and build social capital [6,27], seek feedback [28], and communicate with others [19]. Moreover, Chang and Heo [29] find that user trust in the SNS and their level of activity affects their self-disclosure, especially on Facebook, while Aharony [6] reveals that psychographic and demographic elements such as personality traits and age also affect self-disclosure. Additionally, Cheung et al. [9] opine that social influence stemming from a user observing how other users disclose information about themselves may serve as a catalyst for the sharing of information that may previously have been withheld. This is supported by evidence from Chou et al. [30], which displays that when companies share more information about themselves, their customers also are more open toward them. It is evident, therefore, that although users are generally aware of the privacy concerns on such platforms, their needs to participate in the online community and share of themselves with others often outweigh their fears [31]. 

Furthermore, prior studies on the motivations behind self-disclosure on social networking sites have specified that the interplay of perceived costs and perceived benefits play a significant role. This tallies with social science perspectives of self-disclosure, which hold that individuals weigh costs and benefits before they engage in social exchanges with others. In online contexts, the perceived benefits of self-disclosure include the convenience of maintaining social relationships [32,33], building and developing social capital through new connections [34], the presentation and manipulation of how the user appears to others [4], and entertainment [18]. The perceived costs, as mentioned earlier, largely encompass the loss of privacy [4,22], which may have several adverse effects in the digital as well as physical world [24,26]. These, according to researchers like Cheung et al. [9] and Acquisti and Gross [21], may be mitigated by users’ trust in the SNS and in their fellow users not to abuse the information disclosed.

## 3. Hypotheses Development and Research Model

### 3.1. Flow

Online flow has been described by some researchers as a cognitive state enjoyed during interaction with websites, where there is a “seamless sequence of responses,” making it easy for the user to lose him/herself in the experience of interacting with the platform [3,35]. This helps in improving engagement with the site and its content, especially as it reduces self-consciousness and allows the user to focus his/her attention on the experience s/he is going through. For the purposes of the current study, therefore, flow is assessed through the user’s focused attention on the social networking site, as well as through their interaction with the site. Together, these indicate how flow may lead to increased self-disclosure [36].

### 3.2. Focused Attention (FAT)

A social networking site user may be so engrossed by the content on the site that they are also motivated to share their experiences with others, and thus, engage in self-disclosure behavior [37]. Such focused attention may be attributed to the intentional and effective design of the website [38], as well as to the quality of information/content. Whatever the cause, research supports the assertion that when consumers experience the environment where others are disclosing information about themselves, they are also motivated toward self-disclosure [9]. Indeed, users may become so engrossed by the content they are engaging with that they are inspired to respond or share their own experiences on the platform [3], thus engaging in self-disclosure. Moreover, where such focused attention and flow is perceived to be a result of the excellent design of the website, consumers are more inclined to believe in the performance quality of the service provider, and trust in its ability to safeguard their disclosure [39]. Such a positive view of the provider and trust has also been proven to engender self-disclosure behavior [9]. Further research also establishes that individuals’ self-disclosure increases with their level of activity and usage on the SNS platform [29]. This provides support for the supposition that those who engage on the SNS with a focused level of attention and thus are more involved in SNS activities will disclose more information about themselves than other users whose attention is not so focused on the site.

Thus, the current study proposes that

**Hypothesis 1** **(H1).** Focused Attention has a significant positive effect on Self-Disclosure.

### 3.3. Interaction (FINT)

Moreover, the interactivity afforded by social networking sites has been a key part of their design in order to attract continuous usage [27,36]. Interaction here refers to the extent to which users engage with each other’s divulged information. Through the avenues provided by SNS not only to read content but engage with it, users can be an active part of SNS activity [34]. Consumers may provide instantaneous and multifaceted responses to content provided by others in the form of text, emojis, GIFs, or photos while also enjoying the same immediate kind of feedback from others in their social networks [3]. Such constant interaction provides the opportunity for users to disclose a lot of information about themselves on social networking sites, and thus increase their social capital [28]. Furthermore, the quality and frequency of interaction with others increases the level of trust that the user has in other members of the social networking site [40], and thus achieves the objectives of creating and maintaining social relationships [41]. This serves as a motivation for disclosing more information about themselves and increases their tendency toward self-disclosure. Finally, it has been intimated that consumers who are using the SNS primarily for entertainment and enjoyment purposes tend to disclose more information about themselves [42]. As consumers gain the greatest enjoyment by participating in ongoing conversations within their social spheres [43,44], it is conceivable that users who are engaged in interaction are more likely to disclose more about themselves on social networking sites.

We therefore hypothesize that

**Hypothesis 2** **(H2).** Interaction has a significant positive effect on Self-Disclosure.

### 3.4. Perceived Control (PCL)

Perceived control on SNS refers to how capable and responsible users feel that they are over the sharing of information which they have disclosed. On most social networking sites, some measure of control over the information available to the public is provided. However, consumers may still feel that they still lack final control over what gets seen by whom. Such a perception is exacerbated by reports of SNSs like Facebook and Twitter complying with government requests for personal information on specific users, without their express permission. Meanwhile, the literature provides a premise that when users perceive greater control over their information, they also perceive reduced privacy risk [45], and thus tend more toward self-disclosure [9,46]. Indeed, it becomes a matter of trust in the online service provider, which spurs consumer usage level and self-disclosure [4,47]. Thus, the current study posits that

**Hypothesis 3** **(H3).** Perceived control has a significant positive effect on Self-Disclosure.

### 3.5. Privacy Awareness (PA)

Privacy awareness refers to users’ knowledge and understanding about the options for privacy available to them on a social networking site [9]. While a majority of consumers claim to be aware of how to enforce privacy controls, very few of them actually do so [21]. This has raised questions in research over how aware consumers are about the privacy risks involved in self-disclosure on social networking sites, yielding unsatisfactory results. For instance, only about half of the respondents in some major studies display any concern about their privacy in online environments [48], and a significant proportion of younger social media users remain unaware about privacy risks [25]. Moreover, few users are clear about how the information they disclose on a daily basis is handled or used by the service provider [49], including even those users who claim to have high concern about their privacy [31]. Some studies therefore suggest that among those users who are aware of privacy issues, trust in the SNS and other users reduces, leading to lower levels of self-disclosure. Contrastingly, though, Hoadley et al. [50] and O’Bien and Torres [51] find that some Facebook users with a high level of awareness about privacy issues have increased trust in the service providers and thus share even more about themselves on the platform. This may be an isolated discrepancy in the results due to superior privacy controls instituted on Facebook but could also be attributed to the fact that users with greater awareness of privacy issues maintain or increase their activity levels because they ensure that their connections are secure and that they are relating with people they actually know [52]. It is still then a worthwhile investigation to examine the relationship between privacy awareness and self-disclosure on SNSs. We therefore posit that

**Hypothesis 4** **(H4).** Privacy awareness has a significant positive effect on Self-Disclosure.

### 3.6. Privacy Concerns (PC)

Privacy concerns point out how much a user is concerned about their privacy in online environments [22]. In the literature, fear of cyberbullying, surveillance, stalking, and identity thefts have been cited as key concerns [40,53]. These concerns are often related to the SNS in question and/or the other members of the SNS [4]. Websites and organizations therefore often try to mitigate these concerns by providing facilities like clear privacy policies, which users prefer [54,55], and which help to reduce privacy concerns [56]. Without such interventions, privacy concerns directly affect users’ level of trust in the service provider and inhibit their self-disclosing behaviors [21]. Even so, there seems to be some level of disagreement in the literature concerning the effect of privacy concerns on user disclosure behavior. For example, although Dwyer et al. [57] found that privacy concerns reduce users’ disclosure on Facebook and MySpace, Tan et al. [58] and Boyd and Hargittai [59] found that such privacy concerns do not necessarily affect SNS usage intentions. We seek to therefore discover the actual effect of privacy concerns on self-disclosure, and thus hypothesize that

**Hypothesis 5** **(H5).** Privacy concerns have a significant negative effect on Self-Disclosure.

### 3.7. Privacy Invasion Experience (PIE)

A truism of human behavior is that individuals tend to base their future expectations on past experiences. Thus, where consumers have previously experienced an invasion of their privacy, their trust in the service provider plummets [2]. This is due to the fact that there seems to be an unspoken contract between SNS providers and consumers that their data and information will be kept private. Thus, when there is a breach of this implicit contract, users become disillusioned and lose trust in the provider [60]. Such a negative experience is believed to result in increased privacy concerns and perceived privacy risks [61], making such consumers much more attentive to privacy controls and options for reducing their exposure in online environments. Such consumers are also less willing to provide personal details [61] and may even shut down their accounts on SNS in extreme circumstances [40]. These realities also lead to a decrease in self-disclosure on social networking sites [9], especially compared to other users who have not yet experienced such invasions [61]. However, Li et al. [2] find that where the reputation of the website is positive, users who have previously suffered from privacy invasion experiences may experience reduced perceived risk, which in turn has less of a negative effect on self-disclosure. 

We therefore posit that

**Hypothesis 6** **(H6).** Privacy invasion experiences have a significant negative effect on Self-Disclosure.

### 3.8. Privacy Risk (PR)

Both practice and research reveal that there remains a substantive amount of risk for users of social networking sites. Despite the overwhelming social and business benefits that can be gained by individuals, Aharony [6] cautions that although some people may benefit from self-disclosure on sites like Facebook, others may be at increased risk due to information which they share. Probable risks that users may face include, as noted above, stalking, cyberbullying, sexual solicitation, and internet fraud [31,62]. An appreciation of these risks tends to have a negative effect on self-disclosure [63], and therefore, consumers who perceive a security risk will disclose less information about themselves on social networking sites [4]. The major mitigants to this negative relationship have been identified to be trust and perceived control [9,64]. Interestingly, however, researchers have come across a phenomenon termed as the privacy paradox [31], which occurs in social media contexts but is not replicated in other IS literature. The privacy paradox refers to the willingness of consumers to share large amounts of information about themselves online in order to participate in online communities, despite their general knowledge of the security risks associated with such exposure about themselves. Within other IS contexts, such security risks and privacy concerns would result in decreased activity and usage of the system; however, in social networking sites like Facebook, the reverse is observed.

Therefore, we propose that:

**Hypothesis 7** **(H7).** Privacy risk has a significant negative effect on Self-Disclosure.

### 3.9. Tie Strength (TS)

The strength of the interpersonal ties formed on the social networking site may also have an effect on the level and quality of self-disclosure on the platform [2,6]. Factors related to social capital have been found to affect self-disclosure behavior in SNS environments. For example, Li et al. [2] find that social network size, which is how many friends the user has on the site, strengthens the user’s sense of belonging [65] and thus frees them to disclose more and more of their personal information [2,66], as they feel that they are sharing it with their friends. Moreover, researchers like Petronio [67] suggest that there are certain types of relationships formed on such sites that may engender increased self-disclosure behavior. Illustratively, the user may feel compelled to share more about him/herself if there is a friend on the site who has also previously shared a lot with the user in question. Additionally, where a large proportion of “friends” in users’ connections are existing/offline friends, with whom they feel truly connected, they are more likely to disclose more information about their lives [9], because they feel that they are simply maintaining existing relationships [4]. Again, applications of attachment theory to social media usage have hinted at the fact that some users who suffer from attachment anxiety and/or avoidance make use of SNS connections [68], while those who do not are more inclined to disclose more about themselves on such platforms [69]. Finally, bonding social capital, which highlights close-knit relationships in which emotional support can be shared [41], has been shown to be increased on social networking sites like Facebook [70]. In the presence of such strong social ties, self-disclosure can be predicted.

The current study therefore hypothesizes that

**Hypothesis 8** **(H8).** Tie strength has a significant positive effect on Self-Disclosure.

## 4. Methodology

### 4.1. Measurement Instrument

The current study adopted measures from previous study with the aim of improving content validity [71]. The items were however re-worded to reflect the context of current study. Attention focus was measured with four items adopted from Zhou [72]. Interaction which reflects the extent that users are interacting and supporting each other to share diverse contents through SNS was derived from Lee and Kim [3]. Perceived control was also measured with three items derived from Krasnova et al. [73] and Koufaris [74]. Furthermore, privacy awareness was measured with six items derived from Malik et al. [20]. Privacy concern was measured with items derived from Dinev and Hart [25], while privacy invasion experience was measured with two items adopted from Li et al. [2]. Items for privacy risk were also derived from Martins et al. [75]. Tie strength was measured with three items adopted from Ma et al. [76]. Finally self-disclosure was also measure with items derived from Cheung et al. [9] The constructs used in this study and their corresponding items are listed in the Appendix A. All measurement items were presented in English and measured using a five-point Likert scale anchored between strongly disagree (1) and strongly agree (5).

### 4.2. Sample and Data Collection

In order to test the hypothesized research model, the researchers adopted a survey research methodology to collect data. Data were collected from students in three private universities in Ghana. Students in these universities were sampled based on convenience and handed a paper-based questionnaire. Research assistants were sent to the three universities each with 250 questionnaires Students were first asked if they were under the age of 18, those who answered in the affirmative and were still willing to partake in our survey were given a parental consent form. This form was to be given to their parents to seek their approval before going ahead to fill out the questionnaire. The data were collected over a period of five days. In all, 523 questionnaires were returned, of this number 71 had to be discarded because significant portions of the questionnaires were not filled out. A total of 452 were therefore used for the analysis. From the valid responses, 209 were male and 243 were females. Table 1 shows the profile of our respondents.

## 5. Results and Analysis

Data collected from the survey was analyzed using the Partial Least Square approach to Structural Equation Modelling (PLS-SEM) performed on SmartPLS Version 3 (SmartPLS GmbH, Bönningstedt, Germany). Structural Equation Modelling allowed the researchers to test relationships between latent variables in the proposed research model. The current study adopted the PLS approach since a preliminary study of the data collected showed that the data was non-normal. Also, the PLS approach is more suitable since our model is relatively new and untested. Following the two-step approach to evaluating Structural Equation Model recommended by Chin [77], we first tested the reliability and validity of the measurement model and then went on to test the significance of structural path between the latent constructs in the hypothesized model. 

### 5.1. Measurement Model Assessment

To validate the measurement model, we examined reliability, convergent validity and discriminant validity. The reliability of the constructs was assessed with Cronbach’s alpha and composite reliability. As can be seen from Table 2, both Cronbach’s alpha and Composite reliability values for all constructs are compellingly higher than the 0.7 threshold recommended by Henseler et al. [78]. Convergent validity of the measurement model was assessed using the Average Variance Extracted (AVE). Hair et al. [79] recommend that AVE should be greater than 0.5 for convergent validity to be assured. From Table 2 it can be seen that AVE values for all constructs are greater than the 0.5 threshold, indicative of good convergent validity.

In assessing discriminant validity, the following criteria were used: (a) the square root of the AVE for each construct must be greater than the correlation between that construct and any other construct [80], and (b) the heterotrait-monotrait ratio of correlations (HTMT) values must be less than 0.85 [81]. The results in Table 3 show that the square root of the AVE for each construct is greater than the cross-correlation with other constructs. Also, results of the more recent HTMT (0.85) criterion presented in Table 4 proves discriminant validity. In all, the results showed that the psychometric properties of the measures used in the study were adequate.

### 5.2. Structural Model Assessment

**Figure 1:**
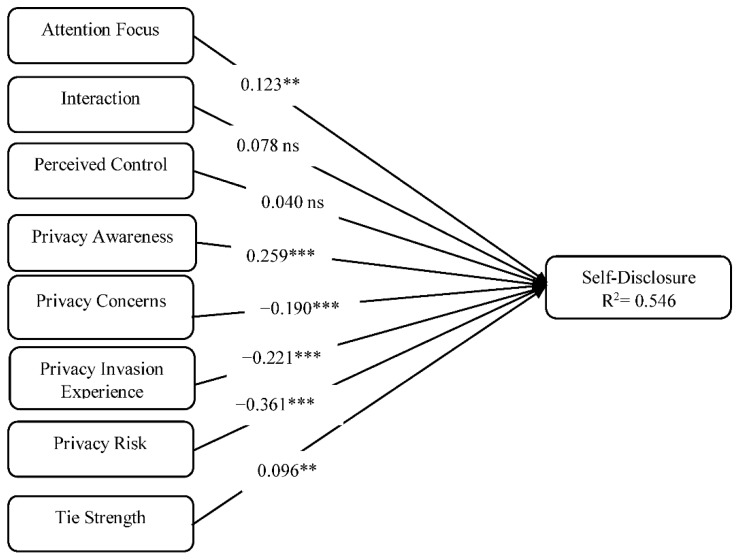
Structural model. *** *p* < 0.001 ** *p* < 0.01 ns—not significant.

Having obtained a satisfactory measurement model, we went on to assess the structural model and determined whether the structural relationships in the model being tested are meaningful. A bootstrap resampling procedure (with an iteration of 5000 sub-samples drawn with replacements from the initial sample of 452) was used to determine the significance of the path coefficients in the structural model. Results for the assessment of the structural model are presented in Table 5 and Figure 1.

In support of H1, focused attention was found to be a significant predictor of self-disclosure (β = 0.123, *p* = 0.006). Interaction and perceived control were found not to be significant predictors of self-disclosure. Privacy awareness was also found to have a significant positive effect on self-disclosure (β = 0.259 *p* = 0.000). Privacy concerns was found to have a significant negative effect on self-disclosure (β = −0.190, *p* = 0.000), thereby providing support for H5. This implies that the more concerned users are about their privacy the less likely they are to self-disclose. Privacy invasion experience was also found to have a significant negative effect on self-disclosure (β = −0.221, *p* = 0.000). This result suggests that users who have had a privacy invasion experience are less likely to self-disclose. Privacy risk was found to have the most significant effect on self-disclosure (β = *−*0.361, *p* = 0.000). Finally, in support of H8, tie strength was also found to have a significant positive effect on self-disclosure (β = −0.096, *p* = 0.004). The authors also performed a multigroup analysis to examine whether there were significant difference in the path coefficients across WhatsApp, Instagram, and Facebook. Results however revealed that there were no significant differences. In all the model accounts for 54.6% of the variance in self-disclosure. The overall fitness of the model was assessed using the SRMR composite factor model. The composite model SRMR value for the model was 0.044, below the 0.08 threshold recommended by Hu and Bentler [82]. This is an indication that the proposed model presents good model fit.

## 6. Discussion

The current study focused on identifying and confirming the factors leading to self-disclosure on various social networking sites, filling an important gap in current literature on the specific predictors of such behavior despite privacy concerns in online environments [31]. The results of the study demonstrate that all hypothesized factors were significant, with the exception of frequent interaction and perceived control. 

Importantly, perceived risk was found to be the most significant predictor of self-disclosure on social networking sites. Previous research has hinted at the negative relationship between the constructs [4,62]; however, studies like that of [31] which illustrate the privacy paradox have called into question the certainty of reduced self-disclosure as a result of privacy risks. The current study contributes to the literature as it indicates that within the Ghanaian context, at least, the privacy paradox may not hold, as users still tend not to disclose as much about themselves when they perceive that their privacy may be at risk. Additionally, other factors related to privacy were found to have the predicted effect on self-disclosure behavior. Although some studies found that a significantly small proportion of SNS users were aware of privacy issues [21], and that those who are subsequently share less about themselves on such sites, our study agrees with research that disputes such a reaction. Like O’Bien and Torres [51] found, users in the current study still share a lot about themselves online even when they are more aware about privacy. Moreover, privacy concerns were also found to have a negative impact on self-disclosure behavior on social networking sites. These findings concur with previous studies [21,57,83,84,85] that demonstrate that user behavior on online social networks is dependent on their perceptions and experience with privacy. SNS users generally expect that that the SNS service provider protects that private data and so when their privacy is abused they are inclined to think that the site did not meet its responsibilities. Users’ past experience often inform their expectations of future encounters when confronted with similar situations. In our study, we found that privacy invasion experience had a negative effect on intention to disclose. This result implies that users who may have experienced an informational exchange-related privacy violation are less like disclose. This result is consistent with those of [86,87], who also found a similar link between privation invasion experiences and behavioral intentions. Our study also found privacy risk to have the most significant effect on self-disclosure. When users disclose their information, they derive benefits such as enjoyment personalization and the strengthening of social ties and this may push them to disclose even more. On the other hand, since some of the information that may be disclosed by SNS may be sensitive, users may be apprehensive of disclosing such information.

The impact of flow characteristics on self-disclosure was also examined by the current study. Focused attention was found to be a significant predictor of self-disclosure, in support of the assumption that when a user is deeply engrossed in the content and quality of the SNS, s/he is motivated to also share about him/herself [9,29]. However, interaction on the SNS was not found to be a predictor of self-disclosure behavior. This contradicts extant studies which find evidence for the relationship [28,42]. It may be understood that when users are enjoying the disclosure of others and interacting with them, they find sufficient fulfilment [44] and find no need to disclose themselves. It has also been speculated that a large number of social media users are more of “lurkers” or “listeners” on social media rather than creators [88,89]; that may account for their enjoyment of others’ disclosure without feeling the need for their own. Also, those who disclose information may be tempted to disclose even more if they realize that whatever they are posting are being viewed by others. In the case of WhatsApp, those who disclose by posting status updates can know who is viewing those updates. Again, perceived control was not found to have a significant positive effect on self-disclosure. This comes as some surprise as other studies have established that when users perceive greater control over their privacy, they perceive reduced risk and thus tend toward self-disclosure [45,46]. Future studies may research whether and which other factors may affect the relationship between perceived control and self-disclosure on SNS.

### 6.1. Implications

The findings of the study provide several useful implications for theory, policy, and practice. The paper has contributed to the literature by introducing a number of factors that serve as significant predictors of self-disclosure on social networking sites in spite of privacy concerns. This provides researchers with a basic relationship upon which future studies can build more complex theories to enhance knowledge in this now-ubiquitous research area. Moreover, policy makers will also benefit from these findings. As it is now evident that privacy issues and concerns have a significant negative impact on the usage and self-disclosure habits of users, regulators must do well to enhance policies which ensure the safety and privacy of the information shared on social networking sites. They must also work closely with SNS creators to prevent exploiters from taking advantage of users’ ignorance on privacy issues and multiply efforts to educate every SNS user to take necessary precautions as they disclose information about themselves.

### 6.2. Limitations and Directions for Future Research

The current study found some exciting result that endorses previous studies, however a few limitations must be taken into consideration when interpreting and generalizing results. First, data were collected from students in three universities in Ghana. Even though this sample represents a fairly typical band of SNS users it is still not representative of all SNS users. Second, our study employed a cross-sectional design, but since user behavior changes over time, it would be interesting to consider a longitudinal design in future studies. Third, the context of our study constrains us from making generalizations to other cultural contexts as well as other stronger economic environments such as advanced economies. Other researchers could consider obtaining data from stronger economic environments and emerging markets and investigating its moderating effect on self-disclosure on SNS. In further studies, other researchers could explore the interrelationship between the factors identified in the current study, for instance the effect the privacy invasion experience could have on privacy concerns [84]. Finally, other researcher could explore the moderating role of gender and age.

## Figures and Tables

**Table 1:** Profile of respondents.

Profile	Measurements	Frequency	Percent
*Gender*	Male	209	46.2
	Female	243	53.8
*Age range*	<18	102	22.6
	18–24	153	33.8
	25–30	119	26.3
	31–40	78	17.3
*Level of Study*	Undergraduate	297	65.7
	Postgraduate	155	34.3
*Privacy settings: my private information is accessible to*	Friends only	131	29.0
	Friends and their friends	103	22.8
	Public	160	35.4
	Don’t know	58	12.8
*Frequency of disclosure*	Several times a day	167	37.0
	Once a day	115	25.4
	Once a week	76	16.8
	Bi-weekly	51	11.3
	Once a month	43	9.5
*N = 452*			

**Table 2:** Results of reliability and convergent validity testing.

Constructs	Items	Loadings	T-Statistics	α	CR	AVE
Attention Focus	FAT1	0.823	32.920	0.854	0.901	0.696
	FAT2	0.852	44.494			
	FAT3	0.849	37.710			
	FAT4	0.811	31.753			
Interaction	FINT1	0.798	24.015	0.835	0.886	0.660
	FINT2	0.802	23.698			
	FINT3	0.782	16.034			
	FINT4	0.864	35.422			
Privacy Awareness	PA1	0.763	30.056	0.888	0.915	0.641
	PA2	0.827	44.898			
	PA3	0.848	50.696			
	PA4	0.806	36.577			
	PA5	0.781	24.422			
	PA6	0.775	29.747			
Privacy Concerns	PC1	0.920	109.744	0.953	0.964	0.843
	PC2	0.937	147.906			
	PC3	0.932	150.543			
	PC4	0.898	61.087			
	PC5	0.903	85.809			
Perceived Control	PCL1	0.875	26.003	0.875	0.922	0.797
	PCL2	0.923	49.418			
	PCL3	0.879	29.125			
Privacy Invasion Experience	PIE1	0.962	223.840	0.918	0.960	0.924
	PIE2	0.960	183.550			
Self-Disclosure	SD1	0.874	73.241	0.891	0.924	0.754
	SD2	0.875	66.567			
	SD3	0.889	78.196			
	SD4	0.833	45.000			
Privacy Risk	PR1	0.953	150.569	0.955	0.971	0.918
	PR2	0.960	219.202			
	PR3	0.961	230.820			
Tie Strength	TS1	0.885	57.270	0.847	0.907	0.765
	TS2	0.851	34.363			
	TS3	0.888	51.252			

**Table 3:** Test of discriminant validity using Fornell-Larcker Criterion.

	FAT	FINT	PCL	PA	PC	PIE	PR	SD	TS
FAT	0.834								
INT	0.614	0.812							
PCL	0.303	0.238	0.893						
PA	0.210	0.195	0.160	0.801					
PC	0.016	0.051	−0.002	−0.206	0.918				
PIE	−0.007	−0.005	0.070	−0.204	0.256	0.961			
PR	−0.002	−0.033	−0.062	−0.134	0.239	0.201	0.958		
SD	0.273	0.251	0.166	0.466	−0.385	−0.393	−0.495	0.868	
TS	0.385	0.364	0.235	0.283	−0.040	0.006	−0.040	0.275	0.875

Note: Square roots of average variance extracted (AVEs) shown on diagonal while off-diagonals are inter-construct correlations.

**Table 4:** Test of discriminant validity using the heterotrait-monotrait ratio of correlations (HTMT) ratios.

	FAT	FINT	PCL	PA	PC	PIE	PR	SD	TS
FAT									
FINT	0.721								
PCL	0.357	0.276							
PA	0.238	0.223	0.178						
PC	0.036	0.068	0.040	0.224					
PIE	0.061	0.043	0.080	0.225	0.273				
PR	0.040	0.077	0.068	0.147	0.248	0.215			
SD	0.312	0.267	0.182	0.523	0.414	0.435	0.536		
TS	0.451	0.425	0.271	0.322	0.050	0.020	0.048	0.313	

**Table 5:** Path coefficients and their significance.

Hypotheses	Hypothesized Path	Path Coefficient	T-Statistics	*p* Values	Result
H1	FAT → SD	0.123	2.736	0.006	Supported
H2	FINT → SD	0.078	1.849	0.064	Not Supported
H3	PCL → SD	0.040	1.292	0.197	Not Supported
H4	PA → SD	0.259	6.543	0.000	Supported
H5	PC → SD	−0.190	6.100	0.000	Supported
H6	PIE → SD	−0.221	6.378	0.000	Supported
H7	PR → SD	−0.361	12.162	0.000	Supported
H8	TS → SD	0.096	2.900	0.004	Supported
Model Fit					
SRMR	0.044				
R-Squared	0.546				

## Appendix A

**Perceived control** Krasnova [73]

PCL1: I feel I am in control over the information I provide on my favorite social networking site
PCL2: Privacy settings allow me to have full control over the information I provide on my favorite social networking site
PCL3: I feel I am in control of who can view my information on my favorite social networking site

**Privacy invasion experience** Li et al. [2]

PIE1: How often have you personally been a victim of what you felt was an invasion of privacy?
PIE2: How much have you heard or read during the last year about the use and potential misuse of information privacy about consumers?

**Privacy awareness**

PA1: I have read the privacy statement of my favourite SNS
PA2: The privacy statement of my favorite SNS is easy to understand
PA3: The privacy settings of my favorite SNS are easy to use
PA4: I understand all the privacy setting of my favorite SNS
PA5: I am aware of all the appropriate actions to ensure my privacy on my favorite SNS
PA6: I am aware of my privacy rights and responsibilities on my favorite SNS

**Privacy concerns** Hart and Dinev [22]

PC1:1 I am concerned that the information I share on SNS could be misused.
PC2:1 I am concerned that a person can find private information about me on social networks.
PC3:1 I am concerned about submitting information on SNS, because of what others might do with it.
PC4:1 I am concerned about submitting information on SNS, because it could be used in a way I did not foresee

**Self-disclosure** Cheung et al. [9]

SD1: I have a comprehensive profile on my favorite social networking platform
SD2: I find time to keep my profile up-to-date
SD3: I keep my friends updated about what is going on in my life through my favorite social networking site
SD4: When I have something to say, I like to share it on favorite social networking site

**Privacy risk** Martins et al. [75]

PR1: The chances of using social networking sites and losing control over the privacy of my personal information is high
PR2: My signing up and using of social networking sites would lead me to a loss of privacy because my personal information would be used without my knowledge
PR3: Internet hackers (criminals) might take control of my account if I self-disclose on social networking site

**Attention Focus** Zhou [72]

AF1: When using my favorite social networking site, I am absorbed intensely in the activity
AF2: When using my favorite social networking site, my attention is focused on the activity.
AF3: When using my favorite social networking site, I concentrate fully on the activity.
AF4: When using my favorite social networking site, I am deeply engrossed in the activity.

**Interaction** Lee and Kim [3]

FINT1: SNS provides much opportunities to participate in communication using online group or communities
FINT2: SNS supports function to make connection with other users
FINT3: SNS helps make swift sharing of thoughts or feelings
FINT4: SNS helps building social relationships with other users

**Tie strength** Ma et al. [76]

TS1: I have good relationships with people in my online social network
TS2: I am in close contact with the people in my online social network
TS3: I enjoy reading news stories shared by the people in my online social network
